# Improving the Development and Implementation of Audit and Feedback Systems to Support Health Care Workers in Limiting Antimicrobial Resistance in the Hospital: Scoping Review

**DOI:** 10.2196/33531

**Published:** 2022-03-11

**Authors:** Julia Keizer, Britt E Bente, Nashwan Al Naiemi, Lisette JEWC Van Gemert-Pijnen, Nienke Beerlage-De Jong

**Affiliations:** 1 Centre for eHealth and Wellbeing Research Section of Psychology, Health and Technology University of Twente Enschede Netherlands; 2 Laboratorium Microbiologie Twente Achterhoek Hengelo Netherlands; 3 Department of Infection Prevention Hospital Group Twente Almelo/Hengelo Netherlands; 4 Section of Health Technology and Services Research Technical Medical Centre University of Twente Enschede Netherlands

**Keywords:** scoping review, audit and feedback, eHealth, development, implementation, antimicrobial resistance, antibiotic stewardship, infection control

## Abstract

**Background:**

For eHealth technologies in general and audit and feedback (AF) systems specifically, integrating interdisciplinary theoretical underpinnings is essential, as it increases the likelihood of achieving desired outcomes by ensuring a fit among eHealth technology, stakeholders, and their context. In addition, reporting on the development and implementation process of AF systems, including substantiations of choices, enables the identification of best practices and accumulation of knowledge across studies but is often not elaborated on in publications.

**Objective:**

This scoping review aims to provide insights into the development and implementation strategies for AF systems for a real-world problem that threatens modern health care—antimicrobial resistance—and provide an interdisciplinary conceptual framework that can serve as a checklist and guidance for making informed choices in the development and implementation of future AF systems.

**Methods:**

A scoping review was conducted by querying PubMed, Scopus, Web of Science, IEEE Xplore Digital Library, and Embase (≥2010) for studies describing either the development or implementation process, or both, of an AF system for antimicrobial resistance or infections in hospitals. Studies reporting only on effectiveness or impact were excluded. A total of 3 independent reviewers performed the study selection, and 2 reviewers constructed the conceptual framework through the axial and selective coding of often-used theories, models, and frameworks (TMFs) from the literature on AF and eHealth development and implementation. Subsequently, the conceptual framework was used for the systematic extraction and interpretation of the studies’ descriptions of AF systems and their development and implementation.

**Results:**

The search resulted in 2125 studies that were screened for eligibility, of which 12 (0.56%); 2012-2020) were included. These studies described the development and implementation processes heterogeneously in terms of study aims, study targets, target groups, methods, and theoretical underpinnings. Few studies have explicitly explained how choices for the development and implementation of AF systems were substantiated by the TMFs. The conceptual framework provided insights into what is reported on the development and implementation process and revealed underreported AF system constructs (eg, AF system design; engagement with the AF system; and comparison, goal setting, and action planning) and development and implementation (eg, champions) constructs.

**Conclusions:**

This scoping review showed the current heterogeneous reporting of AF systems and their development and implementation processes and exemplified how interdisciplinary TMFs can (and should) be balanced in a conceptual framework to capture relevant AF systems and development and implementation constructs. Thereby, it provides a concrete checklist and overall guidance that supports the professionalization and harmonization of AF system development and implementation. For the development and implementation of future AF systems and other eHealth technologies, researchers and health care workers should be supported in selecting and integrating TMFs into their development and implementation process and encouraged to explicitly report on theoretical underpinnings and the substantiation of choices.

## Introduction

### Background

Audit and feedback (AF) is a common reflective approach for various health care targets; however, the reported effects are small to moderate [1]. With the increase in electronically available data in health care, there is great potential for electronic AF systems [2]. The effectiveness of AF systems depends on the targeted behavior and the content, delivery, and context of AF and the system [1,3-6]. These constructs are often studied after AF system development and implementation to evaluate strategies and their ingredients for success [7-10]. In the literature, less attention has been paid to the development and implementation processes of AF systems [3], as is also common in the broader field of eHealth [11,12]. The development process of eHealth can refer to the entire iterative process of developing an eHealth technology, from predesign and design to implementation and (summative) evaluation [13]. However, in this study, we focus on the process from predesign and design (referred to hereafter as development) to implementation, including formative evaluation cycles. This allows us to focus on the early stages of implementation and development that are truly intertwined, as potential implementation issues (eg, limited eHealth skills) should be accounted for early in the development process to avoid well-known pitfalls of stakeholder and context disregard [14]. These phases are entwined by iterative formative evaluation cycles that provide ongoing information on how to improve both the eHealth technology and the development process taking [13].

Development and implementation are essential to gain a profound understanding of relevant stakeholders, their thinking and work processes, and their context (including implementation factors). Without this understanding, a misfit among technology, context, and people is likely to occur, which decreases the effectivity and efficiency of eHealth in practice [13]. It is crucial to consider these constructs from the start of the development and implementation process to avoid common pitfalls in current AF, such as top-down expert-driven audits with feedback at the hospital level rather than personalized, actionable feedback that supports health care workers (HCWs) in improving the quality and safety of health care [15,16].

The application of theories, models, and frameworks (TMFs) is advocated as an integral part of eHealth development and implementation as it identifies what works for whom, why, how, and when, likely resulting in eHealth technology that achieves the desired outcomes [17]. Colquhoun et al [18] and Tuti et al [2] reported that only 9% (n=140) and 29% (n=7) of the included studies in their systematic reviews explicitly used theory to inform AF development and design. Therefore, implicit assumptions about AF working mechanisms and effective AF systems have driven AF development. Although these assumptions might hold true, they were not informed by theory [18,19], whereas there is a clear link between TMFs and eHealth intervention effectiveness [20,21].

To study the development and implementation of AF, this scoping review focuses on a real-world, wicked problem—antimicrobial resistance (AMR). AMR poses an increasing threat to human health and the durability of modern health care [22]. By 2050, AMR is expected to cause more yearly deaths worldwide than cancer currently does [23]. Antimicrobial and diagnostic stewardship programs and infection control programs form an integrated approach of AMR prevention measures (APMs) that aim to reduce and prevent the burden of AMR in hospitals [24]. Previous studies on HCWs’ needs for APM support showed that changing HCWs’ beliefs about their contribution to limiting AMR should be an important aim of APM strategies rather than merely focusing on raising AMR awareness or influencing ad hoc decisions [25,26]. To do so, learning through reflective cycles provides the opportunity to change HCWs’ behaviors by giving them insight into their own behavior and improvement possibilities [15,27]. Therefore, AF for APM (APM-AF) is a promising strategy in the fight against AMR, although it is currently underused and understudied in the field of AMR [7].

There is a clear link between the use of TMFs and APM effectiveness [28-31], and because of the interdisciplinary nature of APM and eHealth, approaches for development and implementation are grounded in a plethora of TMFs [32]. In particular, APM-AF combines behavior change techniques [28-31], participatory eHealth development [33], human-centered and persuasive design [34-37], and improvement [38] and implementation [39] science. Moreover, TMFs have emerged for AF itself (eg, actionable feedback and feedback intervention theory [3-6]) and in the field of AMR (eg, integrated stewardship model [16,24,40]). Combining these TMFs into a conceptual framework that guides the development and implementation of APM-AF is challenging, and there is little guidance on how to create such interdisciplinary conceptual frameworks [41,42].

### Objectives

There seems to be no standardized way of (theoretically) substantiating choices for and reporting on the development and implementation of AF systems, which hinders the identification of best practices and knowledge accumulation [10,43]. Whereas other reviews on AF have mainly focused on the effectiveness of AF systems [1,2], this scoping review focuses on the development and implementation process to further harmonize and professionalize AF system development and implementation. The aim of this study is to gain insight into the development and implementation strategies for APM-AF systems by answering the following research questions:

1. What studies have been conducted so far to study the development and implementation of APM-AF systems?
2. What TMFs are used and described in studies on the development and implementation processes of APM-AF systems?
3. What information has been reported on APM-AF systems, and how are choices substantiated?
4. What information has been reported on the development and implementation processes of APM-AF systems, and how are choices substantiated?
5. What are the lessons learned for the development and implementation of APM-AF systems?

To allow for an evidence synthesis of information on the development and implementation of APM-AF, and because of the explorative aim and research questions in this study, a scoping review is preferred over a systematic literature review [44,45]. This scoping review provides an interdisciplinary conceptual framework that supports researchers, HCWs, and policy makers to make informed choices in APM-AF system development and implementation, with the aim of reducing the burden of AMR and improving the quality and safety of health care.

## Methods

The PRISMA-ScR (Preferred Reporting Items for Systematic Reviews and Meta-Analyses extension for Scoping Reviews) checklist was used to report on this scoping review without a prior registered review protocol [46]. This scoping review was designed by a multidisciplinary research team comprising AMR and eHealth experts.

### Eligibility Criteria

Studies were included if (1) they described the development and implementation process of an AF system (including monitoring and surveillance systems), (2) the system provided feedback to HCWs, and (3) the system targeted AMR and infections in hospitals. A more elaborate description of development and implementation is provided in Multimedia Appendix 1 [13]. We define AF systems as any system that comprises AF, wherein at least one of them (audit or feedback) is delivered or enhanced through the internet and related technologies [47]. As reporting on eHealth development and implementation processes is highly heterogeneous, there were no requirements for specific TMFs, methods, or eHealth technologies. Reviews and poster abstracts were excluded, as were studies outside the hospital setting. Evaluation studies that only reported on APM-AF effectiveness and impact without reporting on development and implementation were excluded. However, constructs of formative evaluation (defined as “activities throughout the entire development process that provide ongoing information on how to improve the development process, outcomes of activities and eHealth technology” [13]) were included, as it is intertwined throughout the eHealth development and implementation process. A full list of eligibility criteria can be found in Multimedia Appendix 1.

### Information Sources, Search, and Selection of Evidence

A comprehensive and systematic literature search in PubMed, Scopus, Web of Science, IEEE Xplore Digital Library, and Embase was conducted without language restrictions. Only studies published in and after 2010 were considered, as both eHealth development and implementation and AMR and APM are rapidly advancing fields. Databases were queried by JK on November 2, 2020, except for Embase, which was queried on January 28, 2021. Together with an information specialist, AMR experts, and eHealth researchers, a structured query was constructed comprising the following terms: *audit* OR *monitor* OR *surveillance* AND *feedback* AND develop* OR implement* AND *infection* OR antib* OR *antimicrobial* OR *resistance*. The results were uploaded to the Covidence web-based software platform (Veritas Health Innovation Ltd), where duplicates were removed. Sources of evidence were selected in a thorough screening process, including title and abstract screening and full-text screening by three researchers independently (JK, BB, and NBJ). After each round, conflicts were discussed until a consensus was reached.

### Data Charting Process

To chart the data, JK created a data extraction form (Multimedia Appendix 2 [2-5,18,43,48-50]) in Microsoft Excel. The general study characteristics extracted were first author, year, journal, country, study aims, targets and target groups, study design and methods, and theoretical underpinning. Given the heterogeneous study approaches and theoretical underpinnings of the included studies, a comprehensive overarching conceptual framework was needed to systematically analyze relevant constructs. The conceptual framework was grounded in often-used TMFs and best practices from various scientific perspectives on AF [3-5,18] and for the description, development, and implementation of eHealth interventions in general [2,43,48-50]. These TMFs and best practices were merged via an iterative axial and selective coding process by JK and NBJ. Thereby, overlapping and complementary constructs from various scientific perspectives were revealed. To structure all constructs, a distinction was made between constructs for APM-AF systems (n=41; research question 3) and constructs for development and implementation (n=35; research question 4).

The data extraction form was discussed within the research team, piloted, and iteratively refined throughout the assessment process. Note that this conceptual framework should be merely seen as an analytic framework for an organized way of thinking about and reporting on APM-AF systems from various perspectives and not as a theory explaining or predicting possible interrelations and outcomes.

### Synthesis of Results

The main researcher (JK) read all full texts and systematically extracted the data using the data extraction form. Studies were scored with a *+* for a comprehensive, *~* for an incomplete, and *-* for a missing description for each data field. Descriptions were copied from the studies and further summarized per data field by describing variations among studies (ie, axial coding). In this process, data fields described by none of the studies were omitted (Multimedia Appendix 2), and other overlapping fields were combined. This reduced the number of data fields for APM-AF systems to 29. The translation and summarization of the extracted data into results were discussed in various rounds within the research team. Owing to the heterogeneity and qualitative nature of the included study designs, the richness and relevance of the (contextual) information were believed to be more important than study quality. Therefore, no quality appraisal was performed [51].

## Results

### Study Selection

**Figure 1:**
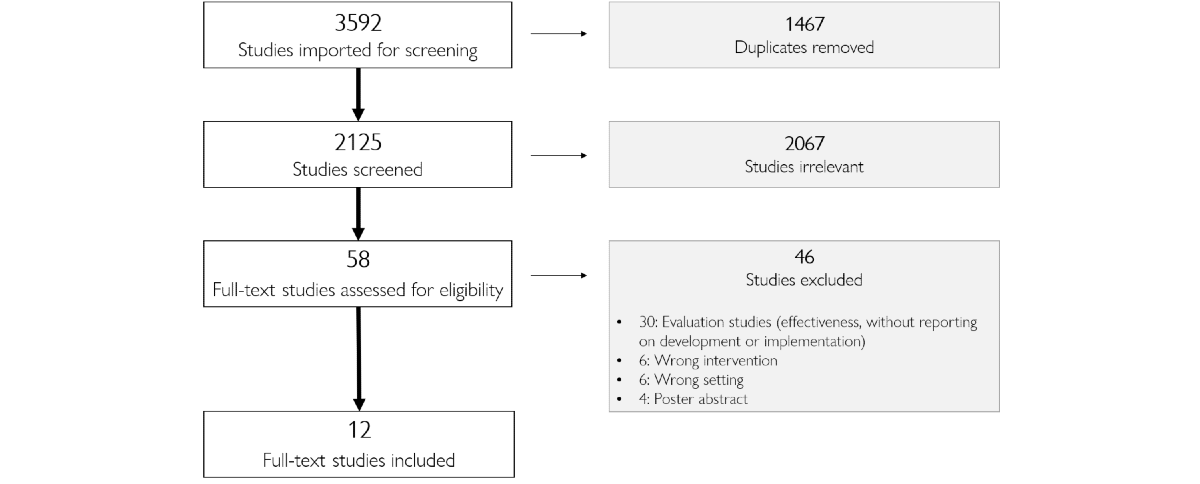
PRISMA (Preferred Reporting Items for Systematic Reviews and Meta-Analyses) flowchart of included and excluded studies, including reasons for exclusion.

The literature search resulted in 3592 potentially relevant abstracts. Of the 3592 papers, after removing 1467 (40.84%) duplicates, 2125 (59.16%) unique titles and abstracts were assessed (Figure 1), which resulted in the eligibility assessment of 58 (1.61%) full texts. The main reasons for exclusion were a lack of information on development or implementation and evaluation studies (without reporting on development or implementation).

### Current State of the Literature Addressing APM-AF Development and Implementation (Research Question 1)

#### Study Characteristics

In total, of the 58 papers, 12 (21%) were included in this review (2012-2020), mostly from PubMed, Scopus, and Web of Science. Publications came from Northern American (6/12, 50%) or European (4/12, 33%) countries and Australia (2/12, 17%). Included studies stemmed from journals in various research fields (eg, infections or implementation science). Studies described APM-AF systems that were either in (preparation of) development or already implemented in practice, resulting in a wide variety of study aims, study targets, target groups, study designs, and used methods (Table 1).

**Table 1:** Study characteristics.

Author and country	Journal	Study aims	Study targets	Target group	Study design and methods	Theoretical underpinning
Boscart et al, Canada [52]	Implementation science	To identify nurses’ and administrators’ perceived barriers and facilitators to current HH^a^ practices and the implementation of a new electronic monitoring technology for HH	ICP^b^ and HAI^c^: HH (improving HH compliance)	Nurses and administrators	Qualitative: Semistructured key informant interviews	Theoretical Domains Framework
Conway et al, United States [53]	The Joint Commission Journal on Quality and Patient Safety	To describe the implementation of an automated group monitoring and feedback system for promoting HH among HCWs^d^ and report its impact on the frequency of HH at a community hospital	ICP and HAI: HH (increase HH frequency)	HCWs (eg, nurses and respiratory therapists), administrators, and managers	Multiple methods: Quantitative: before-and-after study on HH events per patient hour (outcome) Qualitative: focus groups	Model of Actionable Feedback
Edmisten et al, United States [54]	American Journal of Infection Control	To describe the implementation of an electronic HH monitoring system in 3 community hospitals, including the challenges and drivers of success and the maintenance activities needed for continued improvements in compliance with HH practices	ICP and HAI: HH (improving HH compliance)	HCWs, staff, unit/department directors and, facility management	Multiple methods: Quantitative: after study (outcome measures on HH compliance after implementation) Qualitative: direct input from users/department and facility leaders, direct observation, and analysis of system-generated data and sharing of best practices between facilities	None reported
Hysong et al, United States [55]	BMJ Quality and Safety	To describe how feedback intervention theory can be systematically applied in health care settings to design better feedback interventions	DSP^e^ and HAI: to improve internal-medicine resident’s and long-term care personnel’s capacity to distinguish between asymptomatic bacteriuria and catheter-associated urinary tract infection	HCWs (eg, nurse practitioners and staff physicians)	Multiple methods: Quantitative: the Smither et al [56] 11-item scale for recipients’ reactions to feedback Quantitative: chart monitoring (adherence to the treatment algorithm, specifically, rates of urine culture) of orders and inappropriate use of antibiotics	Feedback Intervention Theory
James, Australia [57]	The Journal of Antimicrobial Chemotherapy	To design an audit tool that was appropriate for use in all Australian hospitals, suited to local user requirements, and included an assessment of the overall appropriateness of the prescription	ASP^f^: to improve the quality of patient care by reducing inappropriate and unnecessary use of antimicrobials (national level focus)	HCWs (eg, pharmacists and nurses)	Multiple methods: Quantitative: interrater reliability and validity tests and web-based questionnaire Qualitative: teleconference and direct input from users	None reported
Jeanes et al, United Kingdom [58]	American Journal of Infection Control	To develop and implement an infection control performance and quality improvement data collection tool to meet the needs of large, acute health care providers and improve the credibility and use of infection control performance monitoring	ICP and HAI: to improve the credibility and use of infection control performance monitoring (beyond HH)	Not clearly described; (“auditors” and managers)	Multiple methods: Quantitative: questionnaires and intermittent validation Qualitative: day to day contacts with auditors, feedback from users via the IC-CQI^g^ data input system, discussion groups, and IC-CQI training sessions	Pronovost Knowledge Translation Cycle and Barriers and Mitigation tool, double loop learning cycle, and Hexagon tool framework
Keizer, the Netherlands [59]	Lecture Notes in Computer Science	To describe how a bottom-up participatory development approach can improve the persuasive design of data-driven technologies for their end user (ie, HCWs) and within their context and describe how bottom-up participatory development is a necessary precondition for the development of persuasive data-driven technologies that foster sustainable implementation	DSP, ASP, and ICP: to optimize HCWs’ diagnostic, antibiotic prescription and infection control behavior to limit AMR^h^	HCWs (eg, urologists and residents)	Multiple methods: Quantitative: questionnaire Qualitative: 2 focus groups (last focus group prototype based)	CeHRes^i^ road map
Marques, Portugal [60]	BMC Medical Informatics and Decision Making	To develop a gamification solution that can provide HCWs real-time feedback on personal HH compliance	ICP and HAI: to create awareness regarding HCWs’ HH compliance while trying to change their behaviors and optimize their performance	Nurses	Multiple methods (2 work iterations): Qualitative: preliminary experiments, simulations, field studies, and focus groups	Design Science Research Methodology and gamification
Pakyz, United States [61]	American Journal of Infection Control	To identify the factors related to the implementation of ASP strategies	ASP: to optimize the use of antimicrobial agents, decrease AMR, and decrease rates of *Clostridium difficile* infection	ASP pharmacists and physicians	Multiple methods: Quantitative: survey Qualitative: semistructured telephone interviews	None reported
Parker, Australia [62]	Journal of Clinical Nursing	To provide insights into the experiences of clinicians in implementing a multifaceted bundled urinary catheter care intervention (of which AF^j^ is a considerable component)	HAI: the study aimed to reduce catheter use and duration of catheterization	Clinicians (eg, nurses and resident medical officers)	Qualitative: Postimplementation focus groups	Intervention Description and Replication framework
Patel, United States [63]	Interdisciplinary Perspectives on Infectious Diseases	To describe the development and implementation of their AF intervention using a theoretical framework	ASP: to promote the judicious use of antibiotics	HCWs (eg, neonatologists and pediatric residents)	Multiple methods: Quantitative: retrospective observational study of antibiotic use and clinical vignette study Qualitative: ethnographic workflow study and 2 focus groups	Model of Actionable Feedback
Power, United Kingdom [64]	International Journal for Quality in Health Care	To set up a low-cost pragmatic system to provide monthly data on 4 harms across care settings and produce measures that could be used locally for improvement but also aggregated to determine the burden of harm nationally	HAI: to reduce 4 high volume harms (safety outcomes), pressure ulcers, falls, urinary tract infection in patients with catheters, and venous thromboembolism	HCWs (eg, nurses and junior physicians)	Multiple methods: Quantitative: questionnaire survey (professional satisfaction) Qualitative: paper-based prototyping, formative evaluation by interaction with testers, web forum (including mail queries), regional leads, face-to-face meetings, and regional measurement workshops	ProjectPplan Framework and Plan, Do, Study, Act Method

^a^HH: hand hygiene.

^b^ICP: infection control program.

^c^HAI: hospital-acquired infection.

^d^HCW: health care worker.

^e^DSP: diagnostic stewardship program.

^f^ASP: antimicrobial stewardship program.

^g^IC-CQI: Infection Control Continuous Quality Improvement.

^h^AMR: antimicrobial resistance.

^i^CeHRes: Center for eHealth Research.

^j^AF: audit and feedback.

#### Study Aims

Of the 12 studies, 4 (33%) primarily focused on development, 4 (33%) on implementation, and 4 (33%) described both development and implementation. However, development and implementation appeared to be undefined concepts, with *implementation* studies describing the development and design constructs and *development* studies paying attention to implementation constructs. Studies aimed at describing APM-AF system development focusing on (1) the integration of TMFs (eg, Feedback Intervention Theory), (2) AF content and presentation (eg, feedback gamification), or (3) technical aspects (eg, suitable badges for hand hygiene [HH] monitoring). In addition, studies focused on implementation barriers and facilitators.

#### Study Targets and Target Groups

Of the 12 studies, 11 (92%) focused on one of the APM (ie, diagnostic stewardship programs, antimicrobial stewardship programs, or infection control programs), whereas 1 (8%) targeted multiple APM. The target groups comprised a variety of HCWs (both frontline staff and AMR experts; 8/12, 67%) and in some studies, administrators and managers (4/12, 33%) as well.

#### Study Design and Methods

Most studies (10/12, 83%) used multiple methods, complementing quantitative (eg, questionnaires) with qualitative data (eg, observations, interviews, and focus groups). Approximately 17% (2/12) of the studies were fully qualitative, relying on interviews and focus groups.

### TMFs for APM-AF Development and Implementation (Research Question 2)

#### Theoretical Underpinning Described by Studies

Most studies (9/12, 75%) described the theoretical underpinnings of their APM-AF system or study approach (Table 1). Approximately 17% (2/12) of the studies explicitly mentioned the use of Feedback Intervention Theory and the Model of Actionable Feedback to guide choices in the development and implementation of their study aims [55,63], whereas others mentioned TMFs in their Introduction or Methods section. AF TMFs (3/12, 25%; eg, Model of Actionable Feedback) [53,55,63] were used, as were TMFs, for developing, implementing, and evaluating interventions or technologies (5/12, 42%; eg, Center for eHealth Research road map) [58-60,62,64] and for identifying implementation barriers/facilitators (1/12, 8%; eg, Theoretical Domains Framework) [52]. Substantiations of choices on APM-AF systems were scarce; few studies substantiated their choices, which were either theory informed (eg, providing group-level feedback) or based on findings from the studies themselves (ie, revisions based on formative evaluation).

#### Conceptual Framework for APM-AF Development and Implementation

The conceptual framework, which is based on often-used TMFs and best practices for AF and eHealth interventions, is shown in Table 2 (APM-AF system constructs) and Table 3 (development and implementation constructs) and in Multimedia Appendix 2 in more detail.

**Table 2:** Conceptual framework: APM-AF^a^ system constructs (N=12)^b^.

Constructs and subconstructs				Audit and feedback^c^									eHealth and interventions^d^							Implementation^e^					Studies, n (%)
				[18]		[3]		[5]		[4]		[48]			[43]		[2]		[49]			[50]			
**Audit**																									
	Auditees^f^											✓							✓					10 (83)	
	Main *input*^b^														✓									9 (75)	
**Feedback**																									
	Feedback recipients^f^											✓					✓		✓					8 (67)	
	Main *output*^f^			✓											✓		✓							8 (67)	
	**Level of individualization and specificity**																								
		Feedback provided to individual, groups, or both^f^	✓				✓				✓			✓									11 (92)		
		Feedback is about the individual or team’s own behaviors^b^	✓		✓		✓		✓														10 (83)		
		Feedback level specificity^f^	✓				✓		✓					✓									8 (67)		
	**Comparison, goal setting, and action planning**																								
		Comparison^f^	✓		✓				✓					✓		✓							8 (67)		
		Goal setting^g^	✓		✓				✓							✓		✓					5 (42)		
		Action planning^g^	✓		✓				✓					✓		✓		✓					4 (33)		
	**Feedback framing and incentives**																								
		Punitiveness^b^					✓		✓														6 (50)		
		Attack on self-identity^f^							✓												✓		4 (33)		
		Intrinsic and extrinsic reinforcement or incentives^f^							✓									✓					4 (33)		
	**Timing**																								
		Delivery timing^f^	✓		✓				✓					✓									8 (67)		
		Timeliness (frequency and continuous cycle)^f^	✓		✓		✓		✓		✓			✓		✓							11 (92)		
		Reminders^f^							✓					✓									3 (25)		
**APM-AF system**																									
	**Technology and materials**																								
		Key features of the technology^f^												✓		✓					✓		11 (92)		
		Access^b^												✓									12 (100)		
		Materials^b^									✓			✓		✓							8 (67)		
	**Human–system interaction**																								
		Modes of feedback delivery^f^	✓		✓				✓		✓			✓		✓							9 (75)		
		Level of human involvement^f^												✓							✓		9 (75)		
		Engagement^b^							✓														6 (50)		
	**Design**																								
		Visual presentation strategies and cognitive load^g^	✓		✓		✓									✓		✓					9 (75)		
		User-guided experience^g^	✓		✓		✓		✓		✓			✓		✓		✓					4 (33)		
	**Data validity, d trust and credibility**																								
		Data validity^b^			✓				✓														9 (75)		
		Trust and credibility^f^			✓				✓									✓					11 (92)		
**APM-AF as learning strategy**																									
	**Learning opportunities**																								
		Reflective learning^f^	✓						✓									✓					5 (42)		
		Learning climate^f^							✓									✓					7 (58)		
	Additional strategies or procedures^b^											✓			✓		✓							12 (100)	

^a^APM-AF: audit and feedback for antimicrobial resistance prevention measures.

^b^Unique constructs (ie, where the various perspectives complement each other).

^c^Approximately 72% of constructs theoretically underpinned by literature on audit and feedback.

^d^Approximately 66% of constructs theoretically underpinned by literature on eHealth and interventions.

^e^Approximately 41% of constructs theoretically underpinned by literature on implementation.

^f^Overlapping constructs (constructs represented in 2 perspectives).

^g^Overlapping constructs (constructs represented in all perspectives).

**Table 3:** Conceptual framework: APM-AF^a^ development and implementation constructs (N=12)^b^.

Constructs and subconstructs				Audit and feedback^c^									eHealth and interventions^d^							Implementation^e^					Studies, n (%)
				[18]		[3]		[5]		[4]		[48]			[43]		[2]		[49]			[50]			
**Stakeholders and roles**																									
	Developer or research team^b^											✓												5 (42)	
	Pilot testers and involvement in development and implementation process^f^									✓		✓							✓					11 (92)	
	Leadership engagement^b^																		✓			✓		6 (50)	
	Opinion leaders^b^																		✓					3 (25)	
	Formally appointed internal implementation leaders^b^																		✓					2 (17)	
	Champions^b^																		✓					3 (25)	
**Target behavior and added value**																									
	**Target behavior, problem, and intervention**																								
		Nature of the problem^b^																			✓		12 (100)		
		Description of underlying behavior and decision processes^b^	✓						✓														12 (100)		
		Relevant sociocultural factors and comorbidities^g^																			✓		8 (67)		
		Perceived need for behavior change^g^	✓															✓					4 (33)		
		Targeted behavior is likely to be amenable to feedback^b^	✓		✓				✓														6 (50)		
		Self-efficacy^g^			✓				✓									✓					3 (25)		
		Justify need for behavior change^g^	✓						✓												✓		10 (83)		
	**Rationale and added value**																								
		Rationale for using APM-AF^g^	✓								✓			✓									12 (100)		
		Desirability, efficacy, safety, and cost effectiveness^g^							✓									✓			✓		10 (83)		
		Relative advantage^b^																✓					10 (83)		
**Embedding in practice**																									
	**Implementation complexity and compatibility with target behavior and work processes**																								
		Complexity of implementation process^b^																✓			✓		8 (67)		
		Technology supply model^b^																			✓		8 (67)		
		Compatibility^g^							✓									✓			✓		11 (92)		
		Remove barriers^g^							✓					✓							✓		11 (92)		
		Opportunity costs (including additional efforts to use technology) ^g^							✓									✓			✓		3 (25)		
		Available resources^b^																✓			✓		6 (50)		
	**Inner and outer setting**																								
		Structural characteristics^b^																✓			✓		1 (8)		
		Networks and communications^b^																✓			✓		2 (17)		
		Culture^b^																✓					3 (25)		
		Patient needs and resources^b^																✓					1 (8)		
	**Implementation**																								
		Planning^b^																✓			✓		6 (50)		
		Executing^b^																✓			✓		5 (42)		
**Formative evaluation**																									
	Intended use^b^											✓			✓									1 (8)	
	Actual use^b^											✓												3 (25)	
	Development process and formative evaluations^g^														✓				✓					12 (100)	
	Harms or unintended effects^b^														✓									4 (33)	
	Trialability^b^																		✓					9 (75)	
	Revisions and updating^g^											✓							✓			✓		6 (50)	
	Replicability and digital preservation^b^																		✓					1 (8)	

^a^APM-AF: audit and feedback for antimicrobial resistance prevention measures.

^b^Unique constructs (ie, where the various perspectives complement each other).

^c^Approximately 32% of constructs theoretically underpinned by literature on audit and feedback.

^d^Approximately 24% of constructs theoretically underpinned by literature on eHealth and interventions.

^e^Approximately 74% of constructs theoretically underpinned by literature on implementation.

^f^Overlapping constructs (constructs represented in all perspectives).

^g^Overlapping constructs (constructs represented in 2 perspectives).

The construction of the comprehensive overarching conceptual framework revealed the added value of including multiple perspectives, as 48% of constructs were complementary (ie, covered by one of the 3 perspectives). Overlaps in the coverage of constructs between AF, eHealth, and implementation indicate the integrative nature of the development and implementation of the APM-AF system. Overlapping constructs occurred more often for APM-AF systems (4/29, 14%) than for APM-AF development and implementation (2/35, 6%). In the former, most constructs (21/29, 72% and 20/29, 69%, respectively) came from AF and eHealth literature, whereas in the latter, most constructs (28/35, 80%) came from the implementation literature. Constructs underpinned by all 3 perspectives were not necessarily described by more studies (eg, goal setting; 5/12, 42%).

### APM-AF System Constructs (Research Question 3)

#### Overview

Table 4 shows APM-AF system constructs (more details in Multimedia Appendix 3 [52-55,57-64]). Constructs could be categorized into four main codes—(1) audit, (2) feedback, (3) APM-AF system, and (4) APM-AF as a learning strategy—and are elaborated upon below. Table 4 also shows to what degree and by which studies these constructs were described.

**Table 4:** Constructs of APM-AF^a^ systems (N=12).

Constructs and subconstructs				Described by studies, n (%)							References
				Described elaborately and often substantiated		Partially described or constructed without elaboration or substantiation		Not described or substantiated			
**Audit**											
	Auditees			10 (83)		1 (8)		1 (8)		[52-55,57,59,60,62-64]	
	Main input			9 (75)		3 (25)		0 (0)		[52-55,57,60,62-64]	
**Feedback**											
	Feedback recipients			8 (67)		3 (25)		1 (8)		[53-55,57,59,60,62-64]	
	Main output			8 (67)		3 (25)		1 (8)		[53-55,57-60,62,63]	
	**Level of individualization and specificity**										
		Feedback provided to individual, groups, or both	11 (92)		1 (8)		0 (0)		[52-55,57,59-64]		
		Feedback about the individual or team’s own behaviors	10 (83)		2 (17)		0 (0)		[52-55,59-64]		
		Specificity	8 (67)		1 (8)		3 (25)		[55,59,61,62,64]		
	**Comparison, goal setting and action planning**										
		Comparison	8 (67)		0 (0)		4 (33)		[52,53,55,57,59,60,63,64]		
		Goal setting	5 (42)		1 (8)		6 (50)		[52-54,59]		
		Action planning	4 (33)		3 (25)		5 (42)		[55,58,59,61,62]		
	**Framing and incentives**										
		Punitiveness	6 (50)		0 (0)		6 (50)		[53,55,59-61,63]		
		Attack on self-identity and cognitive influences	4 (33)		0 (0)		8 (67)		[53,58,61,63]		
		Intrinsic and extrinsic reinforcement or incentives	4 (33)		0 (0)		8 (67)		[52,55,60,61]		
	**Timing**										
		Delivery timing	8 (67)		0 (0)		4 (33)		[52,54,60,61,64]		
		Timeliness	11 (92)		1 (8)		0 (0)		[52-55,57-60,62,63]		
		Reminders	3 (25)		0 (0)		9 (75)		[52,53,59]		
**APM-AF system**											
	**Technology and materials**										
		Materials	11 (92)		1 (8)		0 (0)		[52-55,57-60,62-64]		
		Key features of the technology	12 (100)		0 (0)		0 (0)		[52-55,57-64]		
		Access	8 (67)		0 (0)		4 (33)		[53-55,59,60,62-64]		
	**Human–system interaction**										
		Modes of feedback delivery	9 (75)		2 (17)		1 (8)		[52,53,55,59-64]		
		Level of human involvement	9 (75)		3 (25)		0 (0)		[53,55,57-59,61-64]		
		Engagement	6 (50)		0 (0)		6 (50)		[55,59,60,62-64]		
	**Design**										
		Presentation strategies and cognitive load	9 (75)		1 (8)		2 (17)		[52-55,59,60,62-64]		
		User-guided experience	4 (33)		3 (25)		5 (42)		[55,59,60,64]		
	**Validity and credibility**										
		Data validity	9 (75)		1 (8)		2 (17)		[52-55,57,58,60,61,64]		
		Trust and credibility	11 (92)		0 (0)		1 (8)		[52,53,55,57-64]		
**APM-AF as a learning strategy**											
	**Learning opportunities**										
		Reflective learning	5 (42)		0 (0)		7 (58)		[55,59-61]		
		Learning climate	7 (58)		0 (0)		5 (42)		[55,58,59,61,62,64]		
	Additional strategies or procedures			12 (100)		0 (0)		0 (0)		[52-55,57-64]	

^a^APM-AF: audit and feedback for antimicrobial resistance prevention measures.

#### Audit

##### Auditees

The ones audited, or auditees, were described by most studies (10/12, 83%) and comprised frontline HCWs [52-55,57,59,60,62-64].

##### Main Input

Approximately 42% (5/12) APM-AF systems relied on automatically collected input (eg, electronic HH monitoring system) [52-54,60,63], whereas 33% (4/12) systems relied on manual input (eg, audit survey tool) [55,57,62,64].

#### Feedback

##### Feedback Recipients

Feedback recipients were described by most studies (8/12, 67%) and comprised auditees (ie, frontline HCWs) [55,60,62] and managers or administrators [53,54,59,63,64].

##### Main Output

Approximately 67% (8/12) of the studies described APM-AF output in terms of specific content (eg, process, structure, and outcome indicators) [53,54,58,60,62] or provided a general description of output (eg, quality of antibiotic treatment) [55,59,63].

##### Level of Individualization and Specificity

The level of feedback individualization was described by most studies (11/12, 92%). Feedback was provided on individual [55,61] or group level [53,62-64] or on both (ie, option to choose) [52,54,57,59,60]. Approximately 8% (2/12) of the studies explicitly justified their choice to provide group-level feedback only as individual feedback could be perceived as too threatening [53,63]. Feedback was provided to the auditees only [55,60-62], to the auditees and managers or administrators [52,54,59,63,64], or to managers or administrators only [53]. Feedback specificity was described by most studies (8/12, 67%). Feedback was provided on individual patient cases (mostly diagnostic and antimicrobial stewardship studies) [55,61], on aggregated group level (mostly infection control studies) [53,54,60], or on both individual and aggregated levels [59,62,64].

##### Comparison, Goal Setting, and Action Planning

Approximately 67% (8/12) of the studies described data comparison, either in terms of trends over time or benchmarks between groups and with other hospitals [52,57,59,60,63]. Approximately 33% (4/12) of the studies briefly mentioned goal setting and action planning. Goals were either derived from literature [52], based on current data [53,54], or described in terms of an HCW’s need to discuss goals [59] but were not substantiated. Approximately 42% (5/12) of the studies mentioned action planning after feedback, but mostly in general terms (eg, feedback as a tool from which participants could make an actionable plan) [55,61,62], or as a separate follow-up strategy besides the APM-AF system, requiring additional information and human involvement [58,59,61,62].

##### Feedback Framing and Incentives

Some studies mentioned feedback framing in terms of punitiveness (6/12, 50%) or an attack on self-identity (3/12, 25%); however, few specified whether and how these constructs were incorporated in AF system design [53,55,58-61,63]. Approximately 8% (2/12) of the studies incorporated these constructs in their decision to focus only on group-level feedback [53,63], whereas 17% (2/12) studies emphasized nonconfrontational and informal language [60,61]. Intrinsic and extrinsic reinforcement and incentives were addressed in general terms [52,55,61] and more specifically by 8% (1/12) of the studies (eg, competition, win state, and rewards), including how these were implemented in the system (eg, presenting the highest score with a distinct color) [60].

##### Timing

Approximately 42% (5/12) of APM-AF systems made use of feedback at the point of care. This was provided via visual and auditory signals (for HH monitoring) [52,54] or a real-time performance dashboard [54,60,64]. Retrospective feedback was provided in 10 systems, with variable frequencies (daily, monthly, half yearly, and yearly) [52-55,57-60,62,63], sometimes with the possibility of continuously accessing the performance dashboard when needed [53,59,60]. Reminders were mentioned in 25% (3/12) of the studies [52,53,59].

#### APM-AF System

##### Technology and Materials

All studies described their (envisioned future) technologies, which ranged from audit tools (eg, Microsoft Excel or SurveyMonkey [Momentive Inc]) [57,58,61,62,64] to electronic monitoring systems (for HH) [52-54,60], interactive Microsoft PowerPoint presentations [55,63], and prototypes [59]. Access to the APM-AF systems was realized in interactive dashboards or Microsoft PowerPoint presentations with the possibility of sending customized reports via email [54,59,60,64], whereas, in 33% (4/12) of the studies, feedback recipients relied on managers or the research team (via email or face-to-face) for access to the APM-AF system [53,55,62,63].

##### Human–System Interaction

Approximately 17% (2/12) of the studies solely provided feedback via the APM-AF system [60,64], whereas 58% (7/12) of the studies also provided face-to-face feedback [52,53,55,59,61-63]. In addition, studies described the need for additional human involvement, for example, for (educational) meetings with AMR experts [53,57,59,62,63], and support in data processing [55,58,59,61,64]. Half of the studies described how they would engage the user with the APM-AF system via interactive feedback presentations [55,64], gamification [60], or with additional strategies (eg, creating an AF task force) [59,62,63].

##### Design

Design details about included graphs were described by 33% (4/12) of the studies [53,55,60,64], whereas 42% (5/12) of the studies broadly described the APM-AF system design [52,54,59,62,63]. One of the studies used a theory-informed design to ensure that their design matched task and user characteristics [55]. Approximately 33% (4/12) of the studies described an interactive and customizable AF system, wherein personalization was used to customize feedback to match end users’ needs [55,59,60,64]. However, neither was this further specified (eg, which parts are customizable) nor did it focus on user-guided experience (ie, how usability is ensured).

##### Validity and Credibility

Data validity was addressed in terms of raised concerns by study participants [52-54,61], (planned) data validation activities [55,57,58,64], or technical constructs [60]. The trust in and credibility of the APM-AF system was addressed by describing study participants’ perceptions [52,53,58-62] or (planned) activities to improve the trust in and credibility of the system [55,57,63,64].

#### APM-AF as a Learning Strategy

##### Learning Opportunities

Approximately 33% (4/12) of the studies described reflective learning (ie, personal reflections on one’s behavior and APM performance) as a result and strength of APM-AF systems [55,59-61]. Furthermore, APM-AF systems (6/12, 50%) were described as a (potential) facilitator for interactive discussions and communication between HCWs and AMR experts, mostly in existing meetings [55,58,59,61,62,64].

##### Additional Strategies or Procedures

All studies described additional strategies, both for the study (eg, webinar to explain how to use the tool) and for the APM-AF system in practice (eg, creating an AF task force) [52-55,57-64].

### APM-AF Development and Implementation Constructs (Research Question 4)

#### Overview

Table 5 shows the APM-AF development and implementation constructs (Multimedia Appendix 4 [52-55,57-64] provides more details). Constructs could be categorized into four main codes—(1) stakeholders and roles, (2) target behavior and added value, (3) embedding in practice and (4) formative evaluation—and are elaborated upon below. Table 5 also shows to what degree and by which studies these constructs were described.

**Table 5:** APM-AF^a^ development and implementation constructs.

Constructs and subconstructs				Described by studies, n (%)							References
				Described elaborately and often substantiated		Partially described or constructed without elaboration or substantiation		Not described or substantiated			
**Stakeholders and roles**											
	**Stakeholders**										
		Developer or research team	5 (42)		4 (33)		3 (25)		[53,55,57,59,61]		
		Pilot testers and involvement in development and implementation process	11 (92)		2 (17)		1 (8)		[52-55,57,59-64]		
		Leadership engagement	6 (50)		2 (17)		4 (33)		[54,58,61-63]		
		Opinion leaders	3 (25)		0 (0)		9 (75)		[58,61,62]		
		Formally appointed internal implementation leaders	2 (17)		0 (0)		10 (83)		[61,62]		
		Champions	3 (25)		1 (8)		8 (67)		[52,61,62]		
**Target behavior and** **added value**											
	**Target behavior, problem, and intervention**										
		Description of underlying behavior and decision processes	8 (67)		2 (17)		2 (17)		[52,53,55,57-59,62,63]		
		Nature of the problem	12 (100)		0 (0)		0 (0)		[52-55,57-64]		
		Relevant sociocultural factors and comorbidities	12 (100)		0 (0)		0 (0)		[52-55,57-64]		
		Tension for behavior change	4 (33)		1 (8)		7 (58)		[52,53,55,60]		
		Targeted behavior is likely to be amenable to feedback	6 (50)		0 (0)		6 (50)		[52,53,55,60-62]		
		Self-efficacy	3 (25)		0 (0)		9 (75)		[52,53,57]		
		Justify need for behavior change	10 (83)		0 (0)		2 (17)		[52-55,58-62,64]		
	**Rationale and added value**										
		Rationale for using AF^b^	12 (100)		0 (0)		0 (0)		[52-55,57-64]		
		Desirability, efficacy, safety, and cost-effectiveness	10 (83)		0 (0)		2 (17)		[52,53,55,57-62,64]		
		Relative advantage	10 (83)		0 (0)		2 (17)		[52,53,55,57-62,64]		
**Embedding in practice**											
	**Implementation complexity and compatibility with target behavior and work processes**										
		Complexity of implementation process	8 (67)		1 (8)		3 (25)		[54,58-64]		
		Technology supply model	8 (67)		0 (0)		4 (33)		[53,54,58,60-64]		
		Compatibility	11 (92)		1 (8)		0 (0)		[52,54,55,57-64]		
		Remove barriers	11 (92)		0 (0)		1 (8)		[52,54,55,57-64]		
		Opportunity costs (including additional efforts to use technology)	3 (25)		1 (8)		8 (67)		[58,60,62]		
		Available resources	6 (50)		2 (17)		4 (33)		[52,57,58,61-63]		
	**Inner and outer setting**										
		Structural characteristics	1 (8)		0 (0)		11 (92)		[62]		
		Networks and communications	2 (17)		0 (0)		10 (83)		[61,62]		
		Culture	3 (25)		3 (25)		6 (50)		[59,61,63]		
		Patient needs and resources	1 (8)		1 (8)		10 (83)		[54]		
	**Implementation**										
		Planning	6 (50)		0 (0)		6 (50)		[54,58-60,62,64]		
		Execution	5 (42)		0 (0)		7 (58)		[54,58,60,62,64]		
**Formative evaluation**											
	**APM-AF system use**										
		Intended use	1 (8)		1 (8)		10 (83)		[64]		
		Actual use	3 (25)		2 (17)		7 (58)		[58,60,64]		
	Development process and formative evaluations			11 (92)		1 (8)		0 (0)		[52-55,57-60,62-64]	
	Harms or unintended effects			4 (33)		0 (0)		8 (67)		[54,61,63,64]	
	Trialability			9 (75)		1 (8)		2 (17)		[52-55,57,59,60,62,64]	
	Revisions and updating			6 (50)		1 (8)		5 (42)		[52-54,57,60,64]	
	Replicability and digital preservation			1 (8)		1 (8)		10 (83)		[64]	

^a^APM-AF: audit and feedback for antimicrobial resistance prevention measures.

^b^AF: audit and feedback.

#### Stakeholders and Roles

##### Research Team

Approximately 42% (5/12) of the studies described their research team, comprising multidisciplinary stakeholders (eg, HCWs, AMR experts, biostatisticians, and researchers) [53,55,57,59,61]. The research team compositions were substantiated (eg, having a multidisciplinary mix in the team [53,55,57,59,61] and experience with specific research methods [53,55,61]).

##### Pilot Testers and Involvement in Development and Implementation Process

Pilot testers were described by 92% (11/12) of the studies [52-55,57,59-64] and were predominantly selected for their occupational function [52,55,57,59,60,62-64], whereas other details about stakeholders’ expertise and background were hardly described [52,53,57]. Stakeholder involvement was realized by including stakeholders (eg, HCWs and AMR experts) in the research team [62,63] and by involving them in pilot tests and formative evaluations [57,59,60,62-64]. Leadership engagement was mentioned by half of the studies as facilitator for successful implementation [54,58,61-63], whereas stakeholder engagement through champions or opinion leaders was mentioned less often (4/12, 33%) [52,58,61,62].

#### Target Behavior and Added Value

##### Target Behavior, Problem, and Intervention

The nature of the problem and relevant sociocultural factors were addressed by all studies [52-55,57-64]. Most studies (8/12, 67%) provided a description of underlying behavior and decision processes, either shortly in the article’s introduction [53,57] or in a prior study [52,55,58,59,62,63]. Some studies paid attention to whether there was tension for behavior change [52,53,55,60], whether the targeted behavior is likely to be amenable to feedback [52,53,55,60-62], and self-efficacy of feedback recipients’ (ie, feeling capable and responsible for behavior improvement) [52,53,57]. The need for behavior change was justified by pointing out flaws in current behaviors [52-55,58-62,64].

##### Rationale and Added Value

All studies described the rationale and added value of the APM-AF [52-55,57-64]. Approximately 58% (7/12) of the studies described recommendations from health authorities (eg, World Health Organization) or AF as a widely used intervention in health care in general as reasons for developing and implementing an APM-AF system [53-55,57,59,61,63]. Other studies (5/12, 42%) explained how APM-AF could solve problems and inefficiencies in the current situation [52,58,60,62,64]. The added value was described both in terms of expectations (eg, improving the efficiency of audits) and experiences (eg, feedback motivated to change behavior) [52,53,55,57-62,64].

#### Embedding in Practice

##### Implementation Complexity and Compatibility With Target Behavior and Work Processes

Most studies (8/12, 67%) described constructs related to implementation complexity, including required organizational configurations and dependability on suppliers for customizations in terms of expected or experienced implementation barriers [53,54,58-64]. One of the studies specifically reflected on the duration and effort of the entire implementation process [58]. Approximately all studies (11/12, 92%) described constructs regarding the compatibility between the APM-AF system and stakeholders’ needs and existing workflows and expected or experienced implementation facilitators and barriers [52,54,55,57-64]. Opportunity costs were described by a few studies (3/12, 25%) [58,60,62], including negative experiences with the required additional efforts to use the APM-AF (including education and collecting data) [60,62]. A lack of resources was described in terms of staffing, time, and materials [57,58,61-63].

##### Inner and Outer Setting

Few studies (4/12, 33%) paid attention to the inner setting, expressing the need for a collaborative environment and open culture, in which the quality of their work can be discussed safely [59,61,63]. One of the studies described increased patient involvement as a result of visible APM-AF systems [54].

##### Implementation Planning and Execution

Approximately 50% (6/12) of the studies addressed implementation planning, of which 83% (5/6) also reflected on execution [54,58-60,62,64]. Studies mostly reported longer implementation processes than initially planned because of hospital workflow conflicts, personnel availability, and other confounding factors (including technical problems and resistance from stakeholders).

#### Formative Evaluation

##### APM-AF System Use

Intended and actual use of the APM-AF system was hardly (3/12, 25%) described, either as the maximum time HCWs should spend on filling out the audit tool [64] or as how often and complete the audit tool was filled in [58]. In addition, one of the studies used Google Analytics to measure users’ interactions with gamification parts [60].

##### Development Process and Formative Evaluations

The development process and methods used for the formative evaluations were described in all studies [52-55,57-64] and elaborated in this paper’s *Study Characteristics* section.

##### Harms or Unintended Effects

Approximately 33% (4/12) of the studies described whether and how harms and unintended effects were monitored during the development and implementation process (in general terms or with specific examples) [54,61,63,64].

##### Trialability and Revisions and Updating

Approximately 75% (9/12) of the studies described trialability and revisions and updates in terms of several testing rounds [52-55,57,59,60,62,64]. However, only 50% (6/12) of the studies subsequently described, either specifically [57,60,64] or broadly [52-54], how the findings from the testing rounds were incorporated in the design or implementation of the APM-AF system (eg, use of better beacons).

##### Replicability and Digital Preservation

One of the studies published their APM-AF system on the web with additional information (eg, web forum) [64].

## Discussion

### Principal Findings

This scoping review aimed to provide insights into strategies and theoretical underpinnings for AF system development and implementation from a sociotechnical perspective. Of the 2125 studies found in the search, 12 (0.56%) studies were included describing the development and implementation of their AF systems heterogeneously in terms of study aims, AF targets, and development and implementation strategies. Approximately 17% (2/12) of the studies explicitly aimed to illustrate how TMFs could guide choices in AF system development and implementation. A comprehensive interdisciplinary conceptual framework, based on overlapping and complementary constructs from TMFs from AF, eHealth and interventions, and implementation literature, was created to compare the studies.

### Lessons Learned for the Development and Implementation of APM-AF Systems (Research Question 5)

In this discussion, research question 5 is answered by providing lessons learned from reflecting upon our findings for theoretical underpinnings of and reporting on AF, AF systems, and their development and implementation. 

#### Theoretical Underpinnings for AF

With health- and health care–related problems becoming increasingly complex, interdisciplinary theoretical integration to combine different perspectives is inevitable and pivotal to grasp the complexity of real-world problems [65]. This study showed the added value of considering and combining AF, eHealth, and implementation literature for studying AF systems. AF literature covered mostly AF system constructs (21/29, 72%), whereas relevant development and implementation constructs were hardly covered (12/35, 34%). Therefore, studies using only AF literature might miss important development and implementation constructs, such as stakeholder roles (eg, leadership engagement and champions) and embedding in practice (eg, implementation complexity and setting), that influence AF effectivity and efficiency [66,67]. Therefore, we argue that TMFs for AF and for development and implementation should be balanced, as exemplified in our interdisciplinary conceptual framework, and matched with studies’ specific research objectives, methods, and context (eg, setting and participants) [68].

However, selecting and combining the best-fitting TMFs remains a challenge [69]. Well-known examples of combined frameworks exist in implementation science (eg, Theoretical Domains Framework [70]); however, little information is provided about how constructs were combined. Overall, there is little guidance on the selection and integration of interdisciplinary TMFs [71]. Evolving initiatives that create shared languages across disciplines and theories (eg, CohenMiller and Pate [72]) and provide criteria for theory selection (eg, Birken et al [73]) are encouraged.

#### Reporting on AF Systems

This scoping review resulted in an overview of constructs for APM-AF systems (Table 4), enriching the AF best practices from Colquhoun et al [18] with constructs of audit (eg, auditees and audit input), feedback framing and incentives, and AF system constructs (eg, technology and materials, human–system interaction, and data validity and trust and credibility). For replicability and using the framework in actual development and implementation, reporting about the audit input and what technology and materials were used is important. Furthermore, data validity and credibility was deemed as one of the 5 most important aspects for AF in a recent study [42].

In our view, the 2 constructs that were underreported in the included studies require attention in future studies. First, we observed quite broad descriptions of AF system design, with a lack of attention to functional (ie, what can the AF system do) and visual (ie, how efficiently and effectively information is presented to users) design, and engagement with the AF system. However, these constructs are important for how an AF system is received and used in practice [74]. The lack of design aspects and considerations of engagement might reflect the neglect of eHealth system characteristics (such as design) and engagement as active influencers for eHealth effectivity [75]. Second, comparison, goal setting, and action planning were hardly described in the included studies. A lack of substantiations for comparisons was also reported by a review on clinical performance comparators for AF on various clinical topics [76], whereas the underuse of goal setting and action planning was also found by a systematic review on behavior change interventions for APM in hospitals [28]. These 3 constructs were derived from all included theoretical perspectives and are common behavior change techniques [77], suggesting that they require and deserve more attention in future studies.

#### Reporting on Development and Implementation of AF Systems

Of the full-text studies assessed for eligibility, most studies (30/58, 52%) were excluded as they focused primarily on effectiveness and did not sufficiently report on development and implementation. The inclusion of 12 studies in full-text might seem little; however, we believe this is exemplary of the tendency in (APM and eHealth) research to publish more about effectiveness than about the development and implementation process [3,11,12]. Therefore, in future AF system studies, but also for other eHealth technologies, considering development and implementation as influencers of the effectivity and efficiency of eHealth in practice has yet to gain ground [13]. During the construction of the conceptual framework, the interwovenness of development, implementation, and formative evaluation became apparent through the many overlaps observed. This resonates with best practices in eHealth development and implementation, which state that implementation is integrated with development and requires an iterative and holistic approach [78]. Therefore, next to reporting on evaluation, studies should report on both the constructs for AF systems and for development and implementation.

There is no single right development and implementation approach because of the many variations in APM and AF objectives, stakeholders, technologies, and settings [79]. Therefore, the constructed conceptual framework should be seen as a checklist that provides general guidance for potentially interesting constructs to consider; it remains at the discretion of researchers and developers which and how constructs are incorporated in their study. At a minimum, we propose to reflect upon relevant stakeholders and their roles, implementation complexity, compatibility with target behavior, and work processes, including the added value of AF and formative evaluation. Overall, supporting researchers and HCWs in selecting and integrating TMFs into their development and implementation process and promoting the explicit reporting of the theoretical underpinning and substantiation of choices are highly encouraged [41].

The constructed conceptual framework can be used in future studies to ensure a comprehensive view of AF for the transparency and replicability of individual studies. Therefore, we recommend using the conceptual framework as a checklist and adding it (including substantiation of choices) as a supplementary material in future publications. Furthermore, findings from this study can be used to improve the professionalization and harmonization of AF studies, which is important considering the increasing use of AF principles in learning health care systems [80]. The lack of attention to factors that support the development of learning health care systems (eg, organizational culture and cooperation between HCWs and researchers) is recognized as an important barrier to the widespread adoption of learning health care systems [81]. As these aspects are included in the constructed conceptual framework, it might help future researchers and developers to explicitly consider and integrate these constructs in their AF or learning system.

### Limitations and Strengths

This scoping review has several limitations. Although a comprehensive search query was used to search the most important databases for health research, we only included peer-reviewed published research and excluded evaluation studies. As a result, it might be possible that relevant findings were omitted (eg, from gray literature). Two systematic reviews on AF for various health targets were screened to ensure that no relevant publications were missed [1,2]. Another limitation is that evaluation studies were excluded from this review to highlight constructs relevant to the development and implementation, whereas evaluation is critical to know whether an intervention was successful. Therefore, it will be an important next step to evaluate AF systems in terms of processes (eg, improved HH), clinical outcomes (eg, reduced number of infections and decreased AMR), and technological outcomes (eg, APM-AF system use and persuasiveness) [82]. Finally, data extraction relied on the subjective interpretation of the constructs from the included publications by 1 researcher. However, the conceptual framework (Table 2 and Table 3) provided a thorough base for systematic and structured assessment, and the findings were iteratively discussed and revised throughout the review process.

### Conclusions

This scoping review provides novel insights into the theoretical underpinning of and reporting on the development and implementation of AF systems while demonstrating how a comprehensive conceptual framework can be created and used and a valuable means for capturing relevant constructs from heterogeneous studies with varying theoretical underpinnings. Few studies have explicitly described how choices for AF systems and their development and implementation processes were substantiated by TMFs. The interdisciplinary conceptual framework developed in this study is a first step toward the professionalization and harmonization of AF development and implementation with a sociotechnical approach. It provides guidance and a comprehensive checklist to guide researchers, HCWs, and policy makers in making informed choices in the development and implementation of AF systems, with the aim of further improving the quality and safety of health care.

## Appendix

Multimedia Appendix 1 Eligibility criteria.

Multimedia Appendix 2 Data extraction form.

Multimedia Appendix 3 Audit and feedback for antimicrobial resistance prevention measures systems.

Multimedia Appendix 4 Audit and feedback for antimicrobial resistance prevention measures development and implementation.

## Abbreviations

AF: audit and feedback
AMR: antimicrobial resistance
APM: antimicrobial resistance prevention measures
APM-AF: audit and feedback for antimicrobial resistance prevention measures
HCW: health care worker
HH: hand hygiene
PRISMA-ScR: Preferred Reporting Items for Systematic Reviews and Meta-Analyses extension for Scoping Reviews
TMF: theory, model, and framework
