# Supplementary material for: Improving the Development and Implementation of Audit and Feedback Systems to Support Health Care Workers in Limiting Antimicrobial Resistance in the Hospital: Scoping Review
Source: J Med Internet Res. 2022 Mar 11;24(3):e33531. doi: 10.2196/33531 (PMC8957011; doi:10.2196/33531)
Supplement: Multimedia Appendix 1 [file jmir_v24i3e33531_app1.docx]

## Appendix 1. In- and exclusion criteria

Inclusion criteria:

- Development studies (entailing both the predesign (i.e. studying the current and desired situation, how eHealth technology can contribute to resolving issues and identifying the added value, goals, demands and requirements from the context for the potential technology) and design phase (i.e. the actual functional creation of an eHealth technology)) and/or implementation studies (i.e. focused on activities to realize the introduction, adoption, dissemination and long-term use of a product in its intended context), including formative evaluations (i.e. activities throughout the development process that provide ongoing information on how to improve the development process, outcomes of activities and eHealth technology) [13].
- Audit/monitor/surveillance systems
- That provide feedback to healthcare workers (e.g. physicians, nurses)
- Aimed at preventing and limiting antimicrobial resistance and infections

Exclusion criteria:

- Evaluation studies (effectiveness studies, without reporting on development/implementation)
- No information on development/implementation
- Wrong setting (i.e. not in hospital setting)
- Wrong intervention (e.g. AF as minor part of ASP strategy)
- Full-text not available
- Poster abstract (incl. brief reports)
